# Disparity in motorcycle helmet use in Thailand

**DOI:** 10.1186/1475-9276-12-74

**Published:** 2013-08-30

**Authors:** Paibul Suriyawongpaisa, Ammarin Thakkinstian, Aratta Rangpueng, Piyapong Jiwattanakulpaisarn, Pimpa Techakamolsuk

**Affiliations:** 1 Department of Community Medicine, Faculty of Medicine, Ramathibodi Hospital, Mahidol University, Rama VI, Ratchathevee 10400, Thailand; 2 Section for Clinical Epidemiology and Biostatistics, Faculty of Medicine, Ramathibodi Hospital, Mahidol University, Rama VI, Ratchathevee, Bangkok 10400, Thailand; 3 Department of Diseases Control, Ministry of Public Health, Tiwanon, Muang District, Nonthaburi 11000, Thailand; 4 ThaiRoads Foundation, Radploa Rd, Jatujak, Bangkok 10900, Thailand

**Keywords:** Inequity, Helmet use, Resource allocation, Law enforcement

## Abstract

The dispersion of motorcycle related injuries and deaths might be a result of disparity in motorcycle helmet use. This study uses national roadside survey data, injury sentinel surveillance data and other national data sets in 2010 of Thailand, a country with high mortality related to motorcycle injuries, to explore the disparity in helmet use, explanatory factors of the disparity. It also assessed potential agreement and correlation between helmet use rate reported by the roadside survey and the injury sentinel surveillance. This report revealed helmet use rate of 43.7%(95% CI:43.6,43.9) nationwide with the highest rate (81.8%; 95% CI: 44.0,46.4) in Bangkok. Helmet use rate in drivers (53.3%; 95% CI: 53.2,53.8) was 2.5 times higher than that in passengers (19.3%; 95% CI:18.9,19.7). In relative terms (highest-to-lowest ratio,HLR), geographical disparity in helmet use was found to be higher in passengers (HLR=28.5). Law enforcement activities as indicated by the conviction rate of motorcyclists were significantly associated with the helmet use rate (spline regression coefficient = 3.90, 95% CI: 0.48,7.33). Together with the finding of HLR for conviction rate of 87.24, it is suggested that more equitable improvement in helmet use could be achieved by more equitable distribution of the police force. Finally, we found poor correlation (r=0.01; p value = 0.76) and no agreement (difference = 34.29%; 95% CI:13.48%, 55.09%) between roadside survey and injury sentinel surveillance in estimating helmet use rate. These findings should be considered a warning for employing injury surveillance to monitor policy implementation of helmet use.

## Background

Globally, road traffic injuries (RTIs) have been increasing in many regions of the world in contrast to declining trends in highly motorized countries [1]. The majority of RTI related deaths occur in low-income and middle-income countries. Within country, the dispersion of RTIs has also been reported across age groups, gender, economic status and areas [2,3]. Differences in RTIs across areas might reflect lack of access to or scarcity of local goods, services, resources and amenities (area material deprivation) in specific areas as shown by a study investigating the association between area material deprivation/urbanicity and young unlicensed driver involvement in fatal crashes in the United States [4]. At the area level, accumulated evidence supports an association between economic deprivation and low population density and severe RTIs after taking account for the adult or total population of motor vehicle drivers [5,6]. The positive association of young unlicensed drivers involved in fatal crashes in less densely populated areas was demonstrated by a number of studies [7,8].

Concerning reduction of RTIs, the body of evidence supports legislative measures on safety behaviors like helmet use, seat belt use, and compliance to speed limits [9,10]. In addition, White et al. [11] argued that legislative measures are less likely to result in an increase in social inequity in health than those of a voluntary behavioral change nature. RTIs in highly motorized countries mostly involve car drivers, whereas they are motorcycle riders in certain countries of Asia, e.g., Thailand and Vietnam [10,12]. In contrast to the argument of White et al. [11], legislative measures for helmet use applies only on certain assigned routes and national roads in Vietnam which resulted in 6 times the prevalence on inner-city roads [13].

To address inequity in health, monitoring systems were considered part of actions called upon all governments [14]. Lu et al. [15] demonstrated that after passing a mandatory motorcycle helmet law in 1997, the regional inequity in helmet use rate and RTI mortality in Taiwan decreased with a drastic increase in helmet use rate from 45% to 93% during 1997 to 2002 before it leveled off then decreased to 88% 6 years later with resurgence in regional inequity in helmet use rate.

Making use of road-side surveys on helmet use, injury sentinel surveillance and other relevant datasets, this report aims to shed some lights on inequity in helmet use and factors associated with it, and the discrepancy between helmet use rates which were estimated by the survey data and the surveillance data in Thailand.

## Methods

### Helmet use rate

We obtained data on helmet use from 2 sources, which were the nationwide roadside survey [16] and injured surveillance data. The latter were maintained by trauma centers which were assigned to be sentinel sites in 26 provinces of 4 geographical regions across the country [17,18].

Detailed sampling design and data analysis for the nationwide roadside survey were presented in ThaiRoads Foundation [16]. Briefly, the nationwide survey, which was undertaken from May to December in 2010, employed direct observations on helmet use among 945,956 randomly selected motorcyclists (71.4% of which were drivers). These sampled motorcyclists were identified at 3,252 selected sites comprising locations (i.e., road intersections and road sections), and slow traffic in urban and rural municipalities which varied by sizes. The distribution of the selected sites (as a percentage of the total sites for each category) for large, medium, and small municipalities were respectively as follows: 1276 sites (32.9%), 560 sites (17.2%), and 1416 sites (43.5%). Between provinces, the number of sites varied from 22 to 84 according to the area and number of population. For Bangkok, the capital city with a population of 6.9 million [19], 100 sites were randomly selected from 50 districts (2 for each). From this data set, we calculated helmet use rate for the country, each region and each province.

### Contextual and helmet use related factors

Three contextual factors suitable for illustration of disparity in socioeconomic development across provinces were available from the National Socioeconomic and Social Development Board (NESDB). These included percentage of population under poverty lines, gross provincial product (GPP) per capita, and percentage of adult literacy. In addition, two data sets were available for calculating a proxy of traffic law enforcement activities specific to motorcyclists in each province i.e., the number of convicted motorcyclists available from the National Police Office and the accumulated number of registered motorcycles from the Department of Land Transport. Based on the two data sets, we calculated the rate (per 10,000 registered motorcycles) of convicted motorcyclists for each province, hereafter called conviction rate.

### Disparity measures

Health disparity measures are classified into 2 groups, i.e., a relative disparity and an absolute disparity [15]. The relative disparity could be relative concentration index (RCI), rate ratio (RR), and index of disparity (IDisp). The absolute disparity comprised rate difference, between-group variance (BGV), and absolute concentration index (ACI). Each of these measures differs in terms of reference group, whether all individuals in the population are weighted equally, and the scale of measurement of the variable (nominal or ordinal). In practice, making a choice of the disparity measures depends on the availability of data and the scale of measurement.

In this report, we chose highest-to-lowest ratio (HLR) as measures of disparity based on the two practical points and also to allow for comparison with recently published report from Taiwan [15], where their high proportion of motorcycle use was similar to Thailand. To assess disparity in the contextual factors, we calculated the ratio of the highest value to the lowest value. Due to limited space, province specific data were not included but will be available upon request.

### Association between contextual factors and helmet use rate

A cubic spline regression was applied to explore whether relationship between each predictor and the outcome was linear or cubic spline [20]. Data for population density and conviction rate were quite skew and transforming them using natural log-scale also yielded better fit than the raw scales, log-transformation for these 2 variables were thus used. The interested outcome was rate of helmet use whereas the predictors were ln(conviction rate), ln(population density), percent under poverty line, adult literacy, and GPP. These predictors were included in the multivariate spline regression with initial degrees of freedom of 4, and the best knot was then identified for each variable. Deviance statistic with backward elimination was applied to select variables in the model. Model assumption of the final model, i.e., normality of residual, was then checked using Shapiro-Wilk test.

### Difference between helmet use rate estimated by roadside survey and injury sentinel surveillance

The rates of helmet use between the two sources of data were compared for each province. An agreement of the two data sources, hereafter called a gap, along with its 95% confidence interval were estimated using Bland–Altman method [21].

All statistical analyses were performed using STATA version 12.0. A p value <0.05 was considered as statistical significance.

## Results

Table 1 has described relative (HLR) disparity of contextual factors across provinces with relevant figures for each region including Bangkok, the capital city. These included percentage of population under poverty lines, percentage of adult literacy, and rate per 10,000 of convicted motorcyclists (conviction rate). The percentage of population under poverty lines have the biggest magnitude (HLR = 597.70). The next big gap was the conviction rate (HLR = 87.24). Disparity of GPP per capita was shown in relative measure with a ratio of 29.20 between the province with the highest GPP and that with the lowest. The smallest gap was in adult literacy rate (HLR =1.04).

**Table 1:** Contextual factors of helmet use by regions in 2010

**Region**	**No. population (x1000)**	**Poverty line**	**% Under poverty line**	**Population density**	**GPP per capital**	**Adult literacy**	**Convicted motorcyclist**
Bangkok	6876.7	2198	8.0	1330.4	365,619.00	100	38.4
Central	640.1	1725.4	32.6	314.8	246,301.00	99.7	9.2
North	716.2	1572.8	39.8	73.5	78,315.60	98.1	6.3
Northeast	1167.2	1582.7	50.9	132	48,292.10	99.8	6.5
South	669.9	1627	22.6	146.1	113,406.00	99.3	5.9
Inequity across provinces							
HLR			597.70		29.20	1.04	87.24

Table 2 has depicted differences in helmet use rate with 95% CI among combined groups of drivers and passengers and in separated groups across provinces. Helmet use rate was found highest (81.8%) in Bangkok for all types of riders. The relative provincial disparity was highest in passengers (HLR=28.5).

**Table 2:** Prevalence (% ) of helmet use by geographical regions and rider positions including HLR across provinces in 2010

**Region**	**Drivers and passengers**	**N**	**95% CI**	**Drivers**	**N**	**95% CI**	**Passengers**	**N**	**95% CI**
Bangkok	81.8	27,647	81.3,82.2	93.2	21,062	92.8,93.5	45.2	6,585	44.0,46.4
North	37.4	150,888	37.2,37.7	44.8	109,735	44.5,45.1	17.2	41,153	16.8,17.5
North East	38.4	247,821	38.2,38.6	47.6	169,017	47.3,47.8	19.8	78,804	19.5,20.0
Central	53.5	317,301	53.2,53.8	63.9	228,852	63.7,64.1	24.4	88,449	23.7,25.1
South	36	238,946	35.8,36.2	47	167,675	46.7,47.2	9.4	71,271	9.2,9.6
**Nationwide**	**43.7**	**954,956**	**43.6,43.9**	**53.3**	**675,279**	**53.2,53.8**	**19.3**	**279,677**	**18.9,19.7**
Inequality across provinces									
HLR	5.5			4.4			28.5		

Helmet use rates based on injuries sentinel surveillance data set in 2010 (available for 26 provincial sentinel sites) and that from the survey in corresponding provinces were compared, see Table 3. This suggested a gap of 34.29% (95% CI: 13.48,55.09), indicating that the injuries sentinel surveillance data approximately 34% under estimated the use of helmets when compared with the survey data. In addition, the correlation of the two data sets was poor with the estimated correlation coefficient of 0.218 (p-value 0.285).

**Table 3:** Comparison of % helmet use from survey and sentinel injury surveillance in 2010 by province

**Province**	**Male**			**Female**			**Teen**			**Adult**		
	**Survey**	**IS**	**Diff**	**Survey**	**IS**	**Diff**	**Survey**	**IS**	**Diff**	**Survey**	**IS**	**Diff**
Ayutthaya	41.87	12.85	29.02	21.65	13.55	8.10	26.50	11.26	15.24	39.4	13.57	25.81
Saraburi	57.90	17.49	40.41	38.24	11.03	27.21	33.36	10.08	23.28	60.4	17.42	42.98
Chonburi	53.09	1.89	51.20	40.02	1.95	38.07	32.78	0.87	31.91	56.5	2.21	54.33
Rayong	45.33	16.81	28.52	29.85	14.73	15.12	26.02	8.72	17.30	46.4	18.25	28.12
Chanthaburi	35.58	14.61	20.97	27.61	19.62	7.99	21.20	5.32	15.88	39.3	18.56	20.70
Prachinburi	29.23	15.73	13.50	20.98	17.31	3.67	18.32	11.82	6.50	32.1	17.37	14.71
Nakhonratchasima	52.05	25.93	26.12	42.93	31.65	11.28	38.28	17.29	20.99	58.7	31.27	27.44
Surin	59.22	9.99	49.23	39.61	18.99	20.62	43.37	7.88	35.49	70.0	14.1	55.90
Ubornratchathani	49.90	13.69	36.21	32.99	23	9.99	34.27	11.87	22.40	49.0	17	31.98
Khonkaen	54.18	11.93	42.25	11.88	16.04	−4.16	14.28	7.02	7.26	50.3	15.04	35.31
Udornthani	46.13	4.8	41.33	10.71	8.8	1.91	16.19	4.55	11.64	42.2	6.2	35.97
Lampang	44.85	8.45	36.40	47.94	17.35	30.59	29.28	7.08	22.20	55.1	11.7	43.37
Uttaradit	39.73	10.85	28.88	42.17	19.38	22.79	30.60	10.1	20.50	48.7	14.16	34.50
Chiangrai	40.09	4.51	35.58	30.28	9.09	21.19	27.90	2.39	25.51	41.3	6.62	34.69
Nakhonsawan	55.37	15.61	39.76	41.05	17.45	23.60	41.56	10.63	30.93	53.0	17.8	35.20
Phitsanulok	51.41	11.68	39.73	49.63	21.01	28.62	45.47	9.55	35.92	56.5	15.32	41.14
Ratchaburi	31.98	2.53	29.45	22.91	3.42	19.49	21.45	2.66	18.79	35.8	2.85	32.93
Suphanburi	34.74	1.48	33.26	24.57	2.9	21.67	23.36	0.7	22.66	35.3	2.18	33.10
Nakhonpathom	41.28	13.32	27.96	22.05	12.64	9.41	19.69	3.53	16.16	43.1	16.23	26.85
Nakhonsithammarat	42.16	12.29	29.87	29.09	14.82	14.27	16.50	6.99	9.51	44.7	15.34	29.40
Suratthani	37.28	9.66	27.62	31.31	12	19.31	24.60	6.98	17.62	41.9	11.75	30.17
Songkhla	45.51	20.14	25.37	33.75	21.68	12.07	30.77	9.6	21.17	45.0	24.75	20.25
Trang	50.61	8.83	41.78	33.21	10.9	22.31	26.15	4.89	21.26	51.7	11.29	40.40
Yala	25.60	3.25	22.35	25.95	3.37	22.58	13.61	1.31	12.30	30.9	4.14	26.79
Nonthaburi	69.60	5.29	64.31	40.81	1.16	39.65	44.41	4	40.41	71.3	4.64	66.67
Chachengsoa	36.93	6.54	30.39	21.13	5.47	15.66	18.86	2.88	15.98	36.5	7.3	29.15
overall			34.29			17.81			20.72			34.53
95% limiits of agreement*			13.48, 55.09			−2.76; 38.36			3.69, 37.76			12.13, 56.95
rho_c**			0.218			0.332			0.401			0.215
p-value of rho_c			0.285			0.097			0.042			0.292

*Bland & Altman; **Concordance correlation coefficient.

We assessed association between contextual factors and helmet use rate, as described in Table 4.

**Table 4:** Factors associated with helmet use rate according to spline regression analysis

**Use of helmet**	**Coefficient**	**SE**	**t**	**P>|t|**	**95% CI**	
Conviction rate	3.90	1.72	2.27	0.026	0.48	7.33
Population density	6.77	1.49	4.55	<0.001	3.81	9.74

Among 5 predictors (i.e., ln(conviction rate), ln(population density), percent of population under poverty lines, GPP, and adult literacy), only 2 significant predictors (i.e., ln(conviction rate) and ln(population density) were kept in the final model as for a process of model selection. This model met an assumption of normality of residual, which was explored using Shapiro-Wilk test (Z = 1.58, p = 0.056).

Our model suggested that the ln(conviction rate) was linearly correlated with helmet use rate (t = 2.27, p = 0.026) by increasing 1 unit of ln(conviction rate) would increase helmet use rate of 3.9%. Association between ln(population density) and helmet use was also linear, by increasing 1 unit of ln(population density) could increase rate of helmet by 6.8%.

## Discussion

After passage of motorcycle helmet laws for drivers in 1994 and for passengers in 2007 in Thailand, this report revealed helmet use rate of 43.7% nationwide with the highest rate (81.8%) in Bangkok. Helmet use rate in drivers was almost 3 times higher than that in passengers (19.3%). Disparity in helmet use across provinces was found highest among passengers (HLR=28.5). The provincial disparity in 2010 of helmet use in this report was 3 times bigger than that reported in 2008 in Taiwan [15]. The helmet use rate of 88% in Taiwan in 2008 was twice the rate (43.7%) of our study in Thailand. A plausible explanation of these observations is the difference in level of traffic law enforcement between the two countries.

The bigger disparity of helmet use in passengers as compared to that of drivers corresponded with more recent enactment of motorcycle helmet use law for passengers in 2007 than that in 1994 for drivers [22]. Evans indicated larger police forces dedicated to traffic law enforcement were associated with improved automobile driver behaviors [23]. In our study, we found evidence supporting this notion which was indicated by spline regression coefficient of provincial helmet use rate and level of provincial traffic law enforcement represented by ln(conviction rate) of 3.9 (95% CI : 0.48,7.33). (see Table 4). In addition, ln(population density) was associated with helmet use rate with a coefficient of 6.77 (95% CI : 3.81,9.74). Hence, it follows that traffic law enforcement is more feasible in areas with more dense population which facilitates spatial distribution of limited number of police forces.

Considering the disparity in conviction rate across provinces together with the association between the conviction rate/population density and helmet use rate, more equitable improvement in helmet use could be achieved by more equitable distribution of the police force.

This report found no significant association between adult literacy and helmet use rate (see Table 4). This finding could be due to the facts that the relatively low price of motorcycles comparing to 4-wheelers, and the majority of motor vehicles are motorcycles owned by relatively low income households.

This report indicated a big gap (~28%) and poor correlation in helmet use rate between the two sources of data i.e., roadside survey and injury sentinel surveillance. As a result, the injury sentinel surveillance may under estimate the rate of helmet use. This might be due to insufficient funding for sentinel injury surveillance to maintain the quality assurance of data collection [24]; although standardized and uniform hospital data formats have been used since inception of the surveillance [17]. Another explanation is the injury surveillance was mainly focused on hospitalized cases which were more severely injured than non- hospitalized cases indicating a higher probability of not wearing helmet among hospitalized cases [12].

Finally, it is worth mentioning about some limitations of the study. Using cross sectional data, it is difficult to draw a causal association between helmet use rate and those contextual factors found in this report. Although spline regression analysis was applied to test the associations controlling for confounding factors, other known confounding factors such as over speeding, drink driving were not included. Further studies, hence, are needed to better test causal association between helmet use rate and potential predictors.

## Conclusions

Using national representative roadside survey data on helmet use enables us to better demonstrate the disparity in helmet use across provinces as compared to a previous study in Taiwan [15] which relied on hospital-based injury surveillance dataset. We were also able to explore the provincial disparity of helmet use by riding status. With access to national data sets on traffic law policing and socioeconomic status, we were able to explore the potential relationship between helmet use rate and selected contextual factors i.e., conviction rate of motorcyclists, population density, GPP per capita and percentage of residents under poverty line. Finally, we were able to test agreement and correlation between helmet use rate reported by the roadside survey and sentinel injuries surveillance. To the beat of our knowledge, these findings are quite novel.

To achieve equitable and effective allocation of resources for promoting motorcycle helmet use, a country needs to monitor geographical and demographic distribution of the behaviors in the long term. Periodic roadside survey might be considered a monitoring tool for motorcycle helmet use in countries without reliable injury surveillance data, which requires sufficient long term investment.

## Competing interests

The authors declare that they have no competing interests.

## Authors’ contributions

PS drafted the first manuscript and compiled all secondary data sets. AT conducted statistical analyses of the data and reviewed the first draft. AR and PT provided accessed to the injury surveillance data and reviewed the first draft. PJ provided accessed to the roadside survey data and reviewed the first draft. All authors read and approved the final manuscript.

## Authors’ information

Paibul Suriyawongpaisal is an epidemiologist working on road safety policy at national scale for over 2 decades. Ammarin Thakinsatien is a PhD graduate in health statistics with a decade of experiences in applying multivariate analytical methods. Aratta Rangpueng and Pimpa Techakamolsuk are key persons responsible for maintaining the injury surveillance data set at the Ministry of Public Health. Piyapong Jiwattanakulpaisarn has been the principal investigator of the national roadside survey on helmet use conducted annually since 2010.
